# Promoting vaccinations - an analysis of measures taken by German statutory health insurers

**DOI:** 10.1186/2191-1991-1-16

**Published:** 2011-10-04

**Authors:** Kathrin Damm, Jana M Schubert, J-Matthias von der Schulenburg

**Affiliations:** 1 Centre for Health Economics, Leibniz University Hannover, Hannover, Germany

**Keywords:** Vaccination, vaccination rate, health insurance, incentives, promotional measures

## Abstract

**Introduction:**

Prophylactic vaccinations play a significant role in health care. As a relatively cost-effective preventive measure they can help to avert transmissible diseases and thus protect not only the vaccinated individuals themselves but also those who have not been vaccinated. In order to achieve this, a high vaccination rate is necessary; for many prophylactic vaccinations this rate is not reached in Germany. In order to counteract this trend the importance of prophylactic vaccinations was upgraded in 2007 within the scope of the reform of the health system. The reimbursement of patients' vaccination fees was made compulsory for the statutory health insurance companies and statutory requirements were imposed on the insurers to ensure a nationwide provision of prophylactic vaccinations for insured persons. The objective of this paper is to evaluate to what extent the health insurance companies promote the increasing of vaccination coverage rate today and what measures are being used to present this topic to the public.

**Methods:**

In order to assess the public presentation of the topic "prophylactic vaccinations" we have examined the websites of 68 statutory health insurance companies. We have assessed the attitude of the companies towards the promotion of participation in vaccination programs by conducting qualitative, structured interviews with representatives of 8 health insurers.

**Results:**

Measures to promote vaccinations, such as information, recall offers, projects to educate people or even monetary incentives, are employed relatively extensively by the health insurers and are considered important. However, it became clear that the discussion about prophylactic vaccinations, in particular concerning the costs and benefits thereof, had not been completed yet within the companies. Vaccination-specific data is not collected or evaluated on a regular or even isolated basis. There are no concrete targets concerning specific vaccination rates and incentives are neither evaluated nor compared with one another.

**Conclusions:**

The relatively extensive range of measures used to promote the vaccination rate contrasts with insufficient knowledge about their efficacy and efficiency. There is an urgent requirement for more research here.

## Background

Prophylactic vaccinations play a significant role in health care. As a relatively cost-effective preventive measure they can help to avert transmissible diseases and thus protect not only the vaccinated individuals but also those who have not been vaccinated, by containing the spread of pathogenic agents [1,2]. In order to achieve this, a high vaccination rate is necessary which, for many prophylactic vaccinations, is not reached in Germany [3]. The highest rates are amongst children, whose vaccinations can be performed by a pediatrician as part of the regular detection screenings, if parents agree. After childhood this rate drops continuously. For example for diphtheria and tetanus vaccinations the percentage of vaccinated individuals drops from 80 percent in 18 year olds to around 40 percent in 70-79 year olds [4,5].

In Germany vaccinations are usually administered on a voluntary basis; however, the Federal Ministry of Health can order prophylactic vaccinations when there is a danger of epidemics. Reasons for the low vaccination rates are amongst others that infectious diseases are no longer perceived by the population as being a threat due to the success of vaccinations in the past [1]. Further reasons include a lack of information, the inadequate use of doctor-patient contacts and the low importance of preventive medicine [4,5]. At the same time firm convictions, a lack of trust in medical procedures and religious beliefs prevent people from participating in recommended prophylactic vaccination schemes [6].

In 2007 the importance of prophylactic vaccinations was upgraded in Germany. Once the Standing Vaccination Committee (STIKO), the major federal commission concerned with vaccination issues in Germany, recommends particular vaccinations, statutory health insurers were forced to reimburse these. This does not apply to travel or occupational vaccinations. Furthermore statutory requirements were imposed on the insurers to ensure a nationwide provision of prophylactic vaccinations for insured persons.

The objective of this paper is to evaluate to what extent the statutory health insurers promote the increase in vaccination rates and what measures are being used to present this topic in public.

## Methods

In order to assess the public presentation of the topic "prophylactic vaccinations" we examined the websites of 68 statutory health insurers. We took into account publicly accessible insurance companies with at least 100,000 insured. Activities of the health insurers which are suitable for motivating insured persons to participate more in prophylactic vaccination schemes were evaluated. The data analysis was performed using the software SPSS.

In order to examine the general attitude of the health insurers towards the statutory requirements we conducted structured interviews with representatives of eight health insurers of different sizes. We selected representatives who deal with the issue of vaccinations in their companies, in most cases the head of the prevention division. An initial contact has been made via telephone or e-mail.

The questionnaire covered 5 different topics:

1. Possible reasons for the low vaccination rate

2. The situation before 2007:

a. What vaccinations were offered as optional benefits?

b. What was the motivation behind this decision?

c. What was the demand from the insured persons at the time?

d. What incentives were used to motivate the insured persons?

3. The opinion about the legislative amendment and the introduction of prophylactic vaccinations as a compulsory benefit of the insurance companies pursuant to the German Social Code (§ 20 d SGB V)

4. The current situation after the legislative amendment, as for point 2.

5. With regard to "controls":

a. Are vaccination rates recorded?

b. If so, do targets exist?

c. Do sickness funds use measures to increase vaccination rates?

The interviews were actually conducted and recorded by telephone, using internet telephony software ("Skype" Voice over IP client, "Pamela" Call Recorder). The information provided by the interviewees was evaluated with software program MAXQDA, both deductive and inductive [7,8].

## Results

### Analysis of websites

The 68 health insurers included in this analysis insure from 100,000 to more than 7 million individuals. Altogether around 67 million persons are insured by these 68 companies. Because a total of approx. 70 million people in Germany were insured in 202 statutory health insurers in December 2009 [9], 95.7 percent of all these persons are included in the 68 health insurance companies evaluated in our study.

First we examined the way in which the topic of "prophylactic vaccinations" was presented on the websites of the individual insurers. Only for two of the insurance companies no information about prophylactic vaccinations could be found on their websites (see Table 1). All remaining insurers did provide information on their websites. 22 of them presented the information about prophylactic vaccinations which are included in the insurance coverage in a neutral way and explained that it is compulsory for statutory health insurers to meet the cost of prophylactic vaccinations, while the remaining 46 insurers (which corresponds to 67.6 percent) used prophylactic vaccinations as a marketing instrument to a certain extent by emphasizing that they provided this benefit.

**Table 1:** Services of the health insurers to increase vaccination rates

Services of the health insurers	Yes	No	Limited	No information
**Topic "prophylactic vaccinations" on website**	66	2	-	-

**Prophylactic vaccination as a marketing instrument**	46	22	-	-

**Additional information about prophylactic vaccinations**	33	26	9	-

**Provision of a vaccination manager**	22	34	12	-

**Voluntary travel vaccination**	9	23	27	9

**Vaccination campaigns offered**	9	59	-	-

**Bonus or optional programmes with payment for participation**	50	15	3	-

In addition to the general information about the provision of prophylactic vaccinations 33 websites contained further information about prophylactic vaccinations such as vaccination guides with background information about how prophylactic vaccinations work as well as their benefits and risks. Nine websites also include at least some information or references to specialised research facilities. One of the websites provides additional information in a special section for young parents.

The offer of a so-called "vaccination manager" which provides information, includes a planning and reminder function and notifies the insured person registered for this service by e-mail or mail about any upcoming vaccinations, also fits into this context. 22 of the 68 health insurers offer this service in connection with a vaccination calendar which in turn recommends the optimum times for prophylactic vaccinations or booster shots. A similar vaccination calendar was also offered by additional 10 insurers. 34 health insurers did not offer any comparable service.

Some health insurance companies also cover vaccinations for private trips abroad, so-called travel vaccinations. 36 of the 68 insurers advertise these additional vaccinations on their home pages. These are mostly vaccinations against cholera, encephalitis, yellow fever, hepatitis A and B, rabies, typhoid fever, malaria or Japanese encephalitis prophylaxis. 23 health insurers do not cover any costs for travel vaccinations, while nine insurance companies do not mention this topic at all.

Furthermore the campaigns and incentives which the health insurers offer their insured persons on the websites were examined. Special vaccination campaigns were offered by 9 of the 68 health insurers considered, most frequently in the form of advice campaigns or a vaccination telephone number (7 in total).

On the other hand, bonus programmes or elective rates which reward participation in prophylactic vaccinations with gifts, a cash bonus or cost reimbursement are offered by the clear majority of the health insurance companies considered. Of the 68 insurers, 53 offered either a reward programme or optional tariffs with a bonus. Between 1.70 and 20 Euro were offset for individual prophylactic vaccinations; in most cases the bonuses were 10 Euro per vaccination.

### Qualitative interviews

This section presents the results of the qualitative interviews, sorted by topics.

#### Reasons for the low vaccination rate

Three of the interviewees spoke about the reasons for the low vaccination rate. For one expert, this was due mainly to neglect on the part of the insured person. He pointed out that the insured persons usually do not make use of revaccinations or booster shots. This does not point towards an active refusal of prophylactic vaccinations, but more to a failure to return for follow-up visits. Reference was also made to insufficient or wrong information. It was stated that very often the population did not realize how severe a supposedly harmless childhood disease can be, especially if the disease occurs in adulthood, and that the controversial depiction of prophylactic vaccinations in the media had a significant influence on the willingness of the population to be vaccinated. Insured individuals would often allow themselves to be guided by their emotions and less so by facts. Some of them also generally turned down conventional medicine or thought that vaccination recommendations were just a way for pharmaceutical companies to make money. Concerns regarding scientific studies were also voiced. People also suspected the pharmaceutical industry of influencing these studies.

#### Evaluation of the change in legislation

Apart from two interviewees who did not seem to be aware of the legislative change the experts from the health insurers agreed that the introduction of prophylactic vaccinations as a compulsory benefit of the statutory health insurance was an important step in the right direction. The interviewees also nearly unanimously appreciated that all insurance companies now have to offer prophylactic vaccinations nationwide and that decisions about this are no longer left up to the individual insurance companies. The standard, mandatory vaccination catalogue set by the Standing Vaccination Committee (STIKO), which insured persons can use as a guide, would send an important message, namely that vaccinations are good and important. However, for the insurance companies the situation has not in fact changed very much. Even before 2007 the health insurers were already covering all recommended prophylactic vaccinations as optional benefits.

#### Reasons of the health insurers to promote an increase in vaccination rates

Three reasons for the health insurance companies to promote participation in prophylactic vaccinations and to try and increase the vaccination coverage rates of insured persons were named:

1. Six of the interviewees stated that preventive vaccinations made sense from an economic point of view since the costs of an outbreak of a disease were higher than those of prophylactic vaccination.

2. Four health insurer representatives pointed out that it was up to the health insurance companies to make provision for the health of their insured persons to offer them preventive measures and to motivate them to participate.

3. Four of the interviewees also mentioned the competitive situation amongst the health insurers. If some of the insurers cover vaccinations, it was important from a competition point of view to follow suit.

#### Measures to increase the vaccination rate

A number of measures were mentioned as a way of approaching insured persons and increasing awareness about prophylactic vaccinations:

1. General, widespread information: Information about prophylactic vaccinations conveyed to the insured persons in a variety of ways was considered useful by all interviewees.

2. Recall measures: Notifications concerning prophylactic measures needed in the near future were mentioned in three interviews. However, one representative pointed out that only those individuals already interested in the topic could be reached with mailing campaigns.

3. Monetary incentives: Five interviewees spoke about bonus programmes. While three of the interviewees were in favour of bonus programmes as an incentive, two pointed out that the influence of these was limited if people did not want to be vaccinated. Very often only insured persons who would have made use of preventive measures anyway participated in these programmes.

4. Projects for educating insured persons: Five interviewees mentioned projects intended to educate the population about prophylactic vaccinations and prevention. Similar projects have already been carried out in cooperation with health authorities, ministries, and doctors' or pharmacists' associations. These included nationwide vaccination campaigns and vaccination efforts. Particularly for smaller insurance companies, it would be easier to carry out projects together with partners, or to get together and put them into practice on site.

5. Strengthening the doctor-patient relationship: One interviewee mentioned a family doctors' programme in which doctors were specifically required to notify their patients about vaccinations and to motivate them.

#### The role of the health insurers in promoting vaccinations

The influence of the health insurers on the participation of insured persons in prophylactic vaccination schemes was put into perspective by three health insurer representatives, and other health system protagonists who could exert a stronger influence on insured persons were mentioned. Doctors, as medical advisers, were believed to have decisive influence. However, the media also played an important role in shaping the opinion of the patients. The health insurance companies were only mentioned indirectly in the public discussion and could only attempt to take a stand.

#### Controls and evaluation

When asked about the possibilities of collecting and using data about insured persons and vaccination benefits, the responses differed greatly.

The interviewees stated that their companies would not measure the vaccination rate of their insured. They seemed rather surprised about this question. Some of them said that collecting this information was not possible from a technical point of view or could only be achieved with high expenditures. The interviewees had no targets for reaching a certain vaccination rate.

In addition, the experts said that it was not possible to evaluate the measures carried out, since the response rate could not be quantified. Information and campaigns would always be carried out before months with an already high vaccination rate, so it was not possible to determine a trigger for a change in the vaccination rate afterwards. For this reason the measures ran in parallel without a distinction being drawn between their success rates.

All in all, the interviewees did not seem very familiar with the topic of controls with regard to prophylactic vaccinations. Even if it was known that relevant data was being collected or could be collected, there was little or no practical evaluation or use of this data.

## Discussion

To the best of our knowledge, this analysis is the first to examine on the basis of data, the extent to which German health insurers are committed to increasing the vaccination coverage rate and what, if any, measures they use to achieve this.

As the analysis of the websites of the health insurance companies showed, the insurers offer a relatively extensive amount of measures to promote vaccinations and the measures meet the demands of researchers and experts [10]. Apart from general information, this includes recall offers, projects to educate the population and even monetary incentives. However, the results of the interviews in particular but also the current vaccination rates [5] lead to the assumption that these measures are not increasing the participation of the insured person in prophylactic vaccinations to the desired extent. The study results also suggest that there has been little or no change in the efforts and the measures of the health insurance companies as a result of the legislative change. On the contrary, the new legislation which turns prophylactic vaccinations from a competitive factor into a compulsory benefit could even result in the insurers shifting their efforts to other areas which still give them the chance to differentiate themselves.

Nevertheless, the interviews demonstrated that our dialog partners view the increase in vaccination coverage rates as an important step in the right direction. Both the will to ensure the health of the insured persons and to protect them from diseases, as well as the cost issue, acted as incentives to promote an increase in the vaccination rate. However, it became very apparent in the interviews that the discussion about prophylactic vaccinations, in particular their costs and benefits had not yet been completed within the surveyed health insurance companies but that it was generally considered to be important.

Very often, the responses of the interviewees were statements based less on research and facts, and more on educated guesses. It can thus be assumed that there is still a need for information about prophylactic vaccinations in the statutory health insurance companies. There is still a lot of potential particularly in terms of controls, and in the collection and evaluation of data concerning vaccinations. The interviewees revealed that in their companies vaccination rates of insured persons are not measured. Therefore, there is also no attempt to collect informative data or to assess where there are deficits in terms of vaccinations. Furthermore, there are no concrete targets concerning the vaccination rate, and even the individual measures and incentives are not evaluated or compared but tend to be offered somewhat arbitrarily alongside each other. These measures are offered to the insured persons without differentiation. More attention could be paid to the topic of controls in this discussion in order to offer more targeted measures and to generate more information about the acceptance levels and the behaviour of the insured persons.

This evaluation shows that there still seems to be a severe lack of knowledge particularly in terms of explaining the behaviour and motivation of the insured persons when it comes to vaccinations. This is also confirmed by the literature [11,12] and the uncertainty of some interviewees; however, it also leads to the assumption that the insurance companies are not actively promoting an increase in vaccination rates. They would thus show a behavior that Seibt et al. have noticed already among German physicians and pharmacists [13].

In order to reach the goal of increasing vaccination coverage rates, it does not suffice for the insurance companies to just offer incentives; a targeted analysis of the effects of these incentives, which has not been carried out to a sufficient extent yet, is also necessary. For this reason the aim of future studies should be to assess which factors and which actors (e. g. government organizations, insurers, doctors) influence the behaviour of the insured persons to participate in prophylactic vaccination schemes.

Concerning the health insurers, a more active approach as well as the collection and evaluation of data relevant to vaccinations could contribute to bridging the knowledge gaps and to increasing the efficacy and efficiency of the incentives.

The studies carried out here can, of course, only be viewed as first impressions of the current situation. On the one hand the search for measures implemented to increase vaccination rates was restricted to information and data provided on the websites. Although it can be assumed that a large number of the measures are published and promoted here, these details were not assessed. The small amount of interviews held is a further limitation. Only eight interviews could be conducted, resulting in a selection bias. An attempt was made to minimize this limitation as much as possible by specifically selecting relevant distinguishing features (member structure, size, regional or supraregional orientation). It should be noted here that in case of qualitative research psychometric properties like reliability are not in the focal point of interest.

## Conclusions

The health insurance companies promote an increase in vaccination rates by employing a wide range of different measures. However, these efforts seem to contrast with a lack of knowledge about the efficacy and efficiency of these measures. We assume that the significance of prophylactic vaccinations is indeed rated very highly but that the level of information within the health insurance companies does not reflect this. Based on our survey results we can also assume that neither are the vaccination rates of the insured persons recorded nor are the individual measures evaluated and compared. There are also many uncertainties concerning the behaviour and the motivation of the insured persons when it comes to vaccinations. Thus we detect an urgent need for more research in this area.

## Competing interests

The authors declare that they have no competing interests.

## Authors' contributions

KD and JMS were responsible for data acquisition, data analysis and editing. JMvdS reviewed the manuscript.
